# Design of a Single-Tooth Model and Its Application in Oral Scan System Assessment

**DOI:** 10.1155/2021/8891396

**Published:** 2021-03-21

**Authors:** HongXin Cai, Bing Cheng Zhao, Yueyi Tian, Dong-Hyuck Kim, Yunhan Sun, Ho-Kyung Lim, Eui-Seok Lee, Heng Bo Jiang

**Affiliations:** ^1^Stomatological Materials Laboratory, School of Stomatology, Shandong First Medical University & Shandong Academy of Medical Sciences, Tai'an, Shandong 271016, China; ^2^Department of Oral and Maxillofacial Surgery, Graduate School of Clinical Dentistry, Korea University, Seoul 08308, Republic of Korea

## Abstract

Intraoral scanners have been widely used in the application of dentistry. Accuracy includes trueness and precision; they have an important position in the assessment of intraoral scanners. The existing standard models are divided into the inlay and the crown, but the operation is relatively complicated. In this study, in order to simplify the current standard model, we designed a new integration model to compare the accuracy of two intraoral scanners (CEREC and TRIOS) and an extraoral scanner (SHINING). The coordinate measuring machine measured value is the gold standard. Values of the length and angle were analyzed by converting the scanned digital impressions into an STL (standard triangulation language) format to evaluate the accuracy of the intraoral scanner and to verify the feasibility of the designed model. The result shows that the integration model can be successfully scanned and imaged. In the case of the powder-free integration model, intraoral scanner precision, trueness, 3D fitting, and imaging are better than the extraoral scanner. It can be seen straightly from the measurement result and the 3D fitting result that the intraoral scanner can acquire the shape of the standard model integrally with good repeatability. Therefore, it can be concluded that TRIOS is superior to CEREC and SHINING in accuracy, and the integration model is feasible as a reference in the examination of intraoral scanners. The performance of the newly designed integration model that can be scanned is clinically significant, suggesting that this model can be used as a standard reference model.

## 1. Introduction

Computer-aided design and computer-aided manufacturing (CAD/CAM) was introduced into dentistry in the early 1970s [1]. Nowadays, it has been widely used in dental restorations, orthodontic treatments, and the diagnosis of other oral diseases [2–4].

The clinical CAD/CAM system is mainly divided into three parts: the collection of abutment data, the computer designing process, and the milling of the designed model. Among them, digital impression collection plays an important role in this system, which can be divided into direct and indirect methods. Probe standard or optical scanning of traditional dental impressions is an indirect acquisition of abutment data. However, the use of probe standard takes a long time; the large depressions and sharp edges of the impression affect the motion of the probe and thus impair the accuracy of the data [5]. At the same time, the polymerization shrinkage of the impression material, the extrusion deformation, and the collapse of the bubble in the impression material also lead to a decrease in accuracy [6–8]. The intraoral scanner (IOS), as a noncontact direct scanning device, can directly scan the soft and hard tissues. The scanning mechanism mainly includes triangulation calculation, confocal laser scanner microscopy, optical coherence tomography, accordion fringe interferometry, and active wavefront sampling [9–11]. Directly obtained digital impressions in the oral environment can be conveniently stored and analyzed, which is designed to avoid possible errors in indirectly collecting data [12]. Moreover, the intraoral scanner is more convenient than the extraoral scanner, with better patient experience [13, 14]. Consequently, the intraoral scanner has been widely used in clinical practice.

Nevertheless, oral soft tissue, saliva, light, and translucency could lead to an impact on data collection for tooth scans [15–17]. In the meantime, the accuracy would be further attenuated by splicing the scanned images via algorithms or by accumulated rendering errors [5]. Consequently, evaluating the accuracy of intraoral scanners has significant clinical value. According to the definition of accuracy regulated in ISO 5725, which consists of trueness and precision, the trueness refers to the degree of uniformity between a measurement result and the reference value, whereas the latter one maintains the uniformity between independent measurement results. ISO 12836 standard is only restricted to evaluate the accuracy of the extraoral scanner and is not compatible with assessing intraoral scanners. Moreover, there is no well-accepted criterion or device for evaluating the accuracy of intraoral scanners among recent research studies [18].

Flügge et al. indicate that the extraoral scanners perform better than intraoral scanners under the oral environment [19]. However, as the algorithms and scanning techniques evolved, Tomita et al., as they were studying their self-manufactured denture model in vitro, concluded that the intraoral scanners have higher accuracy than extraoral scanners [20]. On the other hand, Ardelean et al. claim disparate conclusions while comparing the results of full denture scans acquired by different brands of intraoral scanners [14].

Whether it is the standard model proposed by the American Dental Association (ADA) or International Organization for Standardization (ISO), it is inevitable to split the standard model into two parts to examine the accuracy of the clinical crown and inlay design. This requires separate scanning of two different standard models, which greatly increases the impact of the environment, the spontaneous deformity of the operator, and the standard model on accuracy [13]. In order to reduce the adverse effects of accuracy on other inspection environments and standard model design issues, we design the standard model to eliminate the influence of the model's own factors on accuracy. The purpose of this study is to design a new integration model and practice it on two intraoral scanners in the clinic as well as an extraoral scanner to evaluate accuracy and feasibility. So, two hypotheses were made in this study. (I) The integration model can be scanned by the intraoral scanner and extraoral scanner. (II) There is no significant difference in the accuracy of the data between the intraoral scanner and the extraoral scanner.

## 2. Materials and Methods

### 2.1. Preparation of the Integration Model

**Figure 1:**
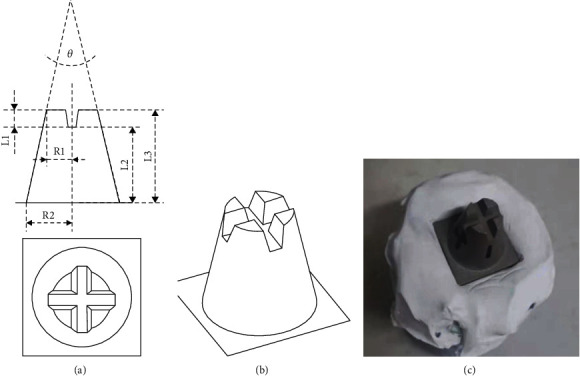
Design and manufacture of the integration standard model. (a) The side and top views with R1, R2, L1, L3, and *θ*. (b) Three-dimensional image. (c) Represents entities of the model.

The 3D file of the integration model was designed by CAD software (AutoCAD 2016), which was exported as an STL format file for CNC milling. The model was made of stainless steel and underwent sandblasting treatment with a powder size of 80 *μ*m. The side and top views of the model and the optical image are shown in Figure 1.

### 2.2. Scanning Process

Two intraoral scanners (CEREC AC D3492, Sirona; TRIOS T12A, 3Shape) and one extraoral scanner (SHINING DS 200+, China) performed 30 scans of the integration model and saved them as STL files. The model was scanned by the same skilled technician to eliminate interference between operators. The operator followed the scanning method recommended by the different instrument manufacturers.

### 2.3. Create Gold Standard Values

In this experiment, the true value of the integration model was measured by CMM (coordinate measuring machine, NC8107, Renishaw, UK), and the CMM measured values and the theoretical values are shown in Table 1. The CMM measurement is set as the gold standard for length and angle evaluation, and a 3D file based on the measured values of the CMM was created as the gold standard file for 3D fitting analysis.

### 2.4. Length, Angle, and 3D Fitting Measurement

Reverse engineering software (Geomagic Control X 2018, 3D Systems, USA) is a measurement tool for measuring relative test indicators (length and angle) of the integration model; the details of the test indicators are shown in Figure 1(a). The 3D fitting measurement via the software compared 3D files between test groups with the gold standard group and recorded RMS (root mean square) values.

### 2.5. Mathematical Analysis

Use the following formula for accuracy assessment:

(1) $$\begin{array}{c} \Delta \text{rA}=\left|\frac{\left(\text{rR}-\text{rA}\right)}{\text{rR}}\right|\times 10^{3}, \end{array}$$

where *Δ*rA is the trueness, rR represents the CMM measurement, and rA represents the actual measured value.

(2) $$\begin{array}{c} \Delta \text{s}\left(\text{rA}\right)=\left|\frac{\text{s}\left(\text{rA}\right)}{\text{rR}}\right|\times 10^{3}, \end{array}$$

where *Δ*s(rA) is the precision and s(rA) represents the standard deviation.

(3) $$\begin{array}{c} \text{RMS}=\left[\frac{\sum {\left(\text{rR}-\text{rA}\right)}^{2}}{30}\right]\frac{1}{2}. \end{array}$$

Perform statistical analysis on scanned data using IBM SPSS v.20.0. The length, angle, and RMS data conform to the normal distribution, but they do not conform to the homogeneity of the variance. The nonparametric Kruskal-Wallis test analyzes the difference in parameters, and the result of *p* < 0.01 attested statistically significant differences.

## 3. Result

In this experiment, we measured the length and radius of the standard model from different aspects. A 95% confidence interval for RMS was obtained by synthesizing the RMS in each independent fit result. The RMS in the 3D fitting results represents the divergence from the contour of the digital reference model, which varies in different scanners and demonstrates their accuracy. The indicator for trueness in this experiment is the calculated true value obtained by analyzing the difference between the measurement results and the reference values and indicating the error of the instrument itself. The variance between measurement results was used to represent the precision, which implies the measurement process random errors. The RMS in the 3D fitting results represents the divergence from the contour of the digital reference model, which varies in different scanners and demonstrates their accuracy.

### 3.1. Analysis of the Length and Angle

**Figure 2:**
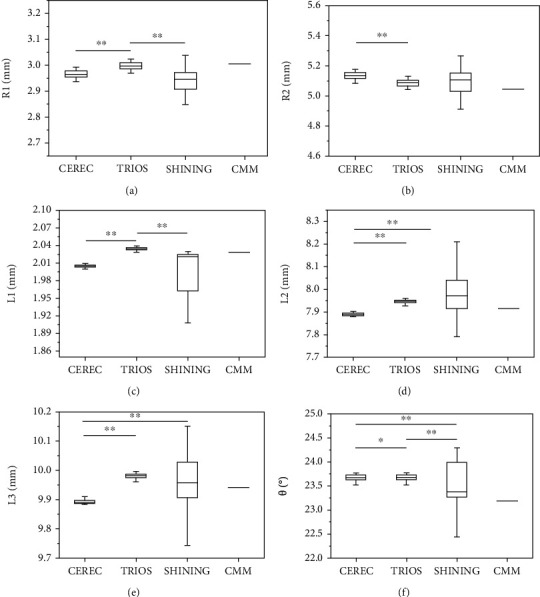
Model measurement comparison (*n* = 30). (a) The average of six parameters of R1. (b) The average of six parameters of R2. (c) The average of six parameters of L1. (d) The average of six parameters of L2. (e) The average of six parameters of L3. (f) The average of six parameters of *θ*. ^∗^*p* < 0.05. ^∗∗^*p* < 0.01. The difference was statistically significant when *p* < 0.05.

In order to study the accuracy of different scanners' lengths and angles, we used CMM to measure the length and angle values of the model as gold standards (Table 1) and compared them with the values measured by the scanner. For comparison, the results are shown in Tables 2 and 3 and Figure 2. The results show that TRIOS is closer to the gold standard than CEREC in terms of R1, R2, L1, L3, and *θ*, and CEREC performs better on L2 than TRIOS and SHINING. The difference between CEREC and the gold standard, particularly the deviation of SHINING in angle *θ*, is obvious with the TRIOS and CEREC references, as shown in Figure 2 and Table 1. In the comparison of the reproducibility of measurement data, the comparison of the precision of CEREC, TRIOS, and SHINING also showed similar results to the true value, and the data reproducibility of TRIOS in L2 (Figure 2(d)) was inferior to that of CEREC. The TRIOS shows that SHINING and CEREC are compared in other data on precision comparisons. Among them, the precision of SHINING is less than that of CEREC.

### 3.2. Analysis of 3D Fitting

**Figure 3:**
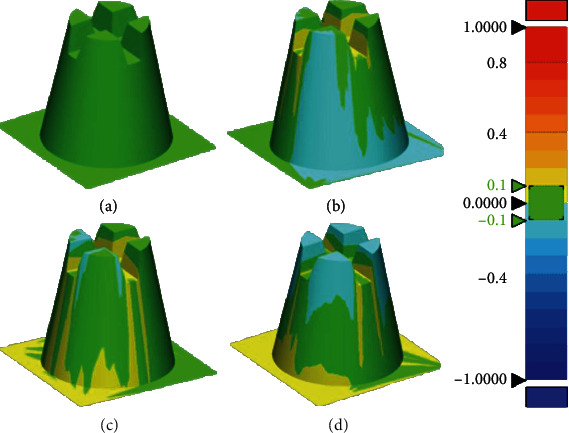
3D fitting comparison of the digital impression. Comparing the digital impression of each scanner system to the gold standard using 3D fitting. (a) Digital impression of the gold standard and images from the 3D fitting file of (b) CEREC, (c) TRIOS, and (d) SHINING.

**Figure 4:**
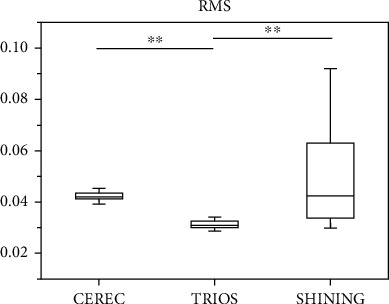
RMS comparison in 3D fitting results (*n* = 30). A box-chart plot of the RMS of various scanner scan results. The horizontal line on the box-chart plot indicates that the RMS is different, and the line end represents this set of data. ^∗^*p* < 0.05. ^∗^*p* < 0.01. The difference was statistically significant when *p* < 0.05.

Figure 3 shows that in this experiment, we converted CEREC, TRIOS, and SHINING scan data into 3D files and overlapped them with 3D files for comparison. The difference in color represents the difference in fitting between the measured value and the gold standard. It shows that SHINING has a larger color difference than other scanners, meaning that the SHINING scan value is much larger than the gold standard. The differences in fitting ranged from TRIOS, CEREC, and SHINING, and their differences ranged from small to large. The TRIOS (0.032 ± 0.008) fitting results are better than CEREC (0.032 ± 0.008) and SHINING (0.0489 ± 0.035). And the RMS value (mean ± SD) of TRIOS (0.032 ± 0.008) for the 3D fitting results is the lowest, CEREC (0.044 ± 0.019) is highly close to SHINING (0.049 ± 0.035). There is no significant difference between CEREC and SHINING (Figure 4).

## 4. Discussion

Digital oral models have been applied to dental restorations, dental implants, and oral and maxillofacial diseases since Duret et al. introduced the CAD/CAM concept into stomatology [21–23]. The oral digital model was created by either intraoral or extraoral methods, where for the latter, a gypsum model should be made for scanning instead of performing chairside scanning. This present research concentrates on studying intraoral scanners for it eliminates the step of preparing the plaster model from the impression material, thus avoiding the deformation or wear of the oral model during transport [6–8]. Afterwards, both the tooth and soft tissue structures were analyzed and modified for preparing a suitable prosthesis for the patient. With the CAM system, the prosthesis can be prepared immediately to restore the prepared tooth functionally with excellent esthetic characteristics, avoiding the laborious sequence including impression taking and laboratory manufacturing.

Accordingly, creating a digital oral model with accurate dimension measurement and adequate detail expression is a chief consideration for further CAM processes. At the same time, the current ISO 12836 for extraoral scanners is not compatible with the intraoral scanners. Current research studies were mainly based on the ADA-relevant standards and formulas in which two separate models for inlay and crown assessment are required. Using multiple standard models in combination increases the uncertainty in scanning and the burden on operators, which may affect the reliability of results. Here, an integration model conforming to the standards of existing standard models is hence created to reduce the impact of the uncertain factors shown above [13].

This standard model is designed with reference to the mandibular first molar by mimicking its dimensional parameter, which is presented in dental clinical practice most frequently. Two perpendicular ditches on the occlusal surface are designed to resemble the prepared MOD cavity with a reasonable depth and tipping angle. According to the model idea provided by ADA, the inlay model and the crown model are redesigned to form an integration model [24]. A concern for repeatability is also demonstrated in the simplified design as the central symmetrical contour, where the least milling process is required. All lateral and inner surfaces are designed with a slight inclination towards the body part of the model to ensure no axial undercut is presented. The integration model was milled from stainless steel in order to provide maximum integrity and strength against abrasion and deformation.

**Figure 5:**
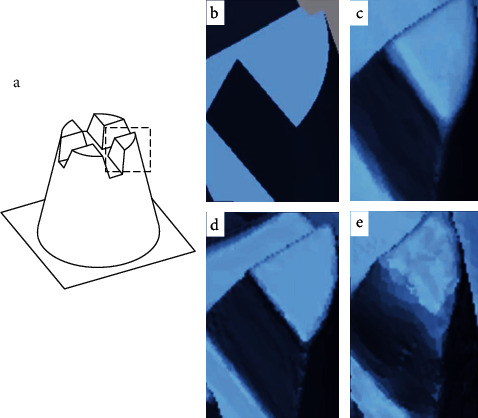
Comparison of digital impressions. (a) Magnify the part of the digital impression and images from 3D files of (b) the gold standard, (c) CEREC, (d) TRIOS, and (e) SHINING.

In this experiment, we measured the length and angle of the standard model from different aspects. As the RMS results showed, the accuracy of TRIOS was generally superior to CEREC and SHINING (Figure 4). In the qualitative analysis of the 3D fitting results, TRIOS also showed a more subtle morphology in contrast to the CMM gold standard (Figure 3). For the details of the digital model, the TRIOS scan showed a smoother surface and a sharper edge (Figure 5). For the results of differences in the height, angle, and radius (L1, L3, *θ*, R1, and R2), TRIOS obtains superior accuracy than CEREC and SHINING. However, CEREC performs better than TRIOS in assessing the depth of the model (L2), suggesting that CEREC was more suitable for inlay restorations than TRIOS, in which more accurate measurement of the cavity depth is required.

The existing errors result from multiple factors. Arakida et al. found that digital models of the highest accuracy can be obtained with an ambient light source of 500 lux and 3900 lux intensity [25]. As for the scanning light, both TRIOS and CEREC use white light which consists of multiple wavelengths, while SHINING uses blue light. For white light, different wavelengths have to be corrected by the specially designed lens in the scanner, as they are not totally refracted at the same focus. However, blue light is more anti-interference than white light due to its shorter wavelength, which can improve the accuracy of the data [26]. Accordingly, Jeon et al. believe that blue-ray scanners are able to scan finer structures and reduce errors [26, 27], whereas this study concluded that the accuracy of the blue-ray scanner SHINING was significantly lower than that of the two intraoral scanners for the following possible reasons. First, in order to maintain consistency in the experiment, the model was powder-free. Whether or not dusting is required depends on the technical principle of the scanning system. The existing scanning technology has two objectives: one is to change the light reflection characteristics of the tooth surface to obtain an excellent diffuse reflection effect, and the other is to artificially increase the surface characteristic information of the tooth and improve the accuracy of the scanning software algorithm. Stainless steel without powder has a high refractive index for light, which affects the scanning sensor's acquisition of refracted light [28]. Based on the technical principle of the scanner, the intraoral scanner uses CLSM (confocal laser scanning microscopy) technology, and the extraoral scanner uses AFI (accordion fringe interferometry) technology. Compared to CLSM, AFI technology uses interference patterns generated by multiple laser sources to produce praiseworthy interference fringes on the target object. However, spots produced by coherent radiation in the image can cause uncertainty in the measurement of the fringe position and limit the range resolution [29]. The intraoral scanner using CLSM technology therefore exhibits better accuracy in scanning rough surfaces. Second, the operator's proficiency also affects the general accuracy of the scanner [30]. The scanning sequence of the scanning operator and the involuntary shaking of the hands during scanning are also factors that affect the accuracy of the scanning. Additionally, the application of different algorithms in different digital model analysis software programs also influences the accuracy to some extent, especially in the process of 3D coordinating and structure merging. After the first image was taken, all subsequent images were stitched to the previous image by the best fitting algorithm, which represented the best possible overlap of the two images. Each overlap had an inherent error, and the final error increases with the stitching process. Therefore, it can be expected that the larger the scanning range was, the more the stitching process was completed, and the greater the error was. Therefore, the splicing algorithm that comes with different scanners may be one of the willingness to make the scanner different in accuracy [31].

All three scanners can better scan the specific shape of the integration model, and the scan values are similar to the gold standard. The repeatability of the study can be proved by the consistency in measurement results and the detailed surface acquired in 3D perspectives. The intraoral group scan bias is less than or close to the standard specified by the ADA. Moreover, the scanning performance of the intraoral scanner clearly showed that the model is thoroughly scanned and the results are not excessively deviated from the gold standard.

## 5. Conclusions

A new integration model has been furnished through this experiment, which can be successfully applied to intraoral scanners and extraoral scanners. Accordingly, we can draw two conclusions in this experiment:

1. The powder-free integration model has great compatibility for evaluating different types of scanners
2. For this integrated model, the intraoral scanner is closer to the true value than the extraoral scanner. The digital impression image of the intraoral scanner is more realistic than the extraoral scanner

In general, the powder-free integration model demonstrates the ability to assess oral scanning systems; and in practical applications, the intraoral scanner is closer to the gold standard than the extraoral scanner. This study reached the goal of evaluating the oral scan system.

## Figures and Tables

**Table 1:** Theoretical and CMM values measured for six scanned indexes.

	R1 (mm)	R2 (mm)	L1 (mm)	L2 (mm)	L3 (mm)	*θ* (°)
Theoretical	3.000	5.000	2.000	8.000	10.000	23.000
CMM	3.003 ± 0.163	5.044 ± 0.226	2.029 ± 0.154	7.914 ± 0.202	9.942 ± 0.158	23.199 ± 4.294

**Table 2:** Precision of six scanned indexes by the three scanners.

Test group	*Δ*rR1	*Δ*rR2	*Δ*rL1	*Δ*rL2	*Δ*rL3	*Δ*r*θ*
CEREC	7.398	6.990	1.430	0.844	1.137	17.944
TRIOS	4.806	4.416	1.890	3.677	0.868	2.566
SHINING	27.072	23.963	36.799	12.746	12.194	31.581

**Table 3:** Trueness of six scanned indexes by the three scanner systems.

Test group	*Δ*sR1	*Δ*sR2	*Δ*sL1	*Δ*sL2	*Δ*sL3	*Δ*s*θ*
CEREC	13.527 ± 14.500	16.972 ± 11.384	11.302 ± 2.804	3.300 ± 1.654	5.076 ± 2.228	24.000 ± 35.170
TRIOS	3.703 ± 6.352	7.837 ± 8.557	2.976 ± 2.591	4.561 ± 4.458	3.935 ± 1.702	3.312 ± 4.489
SHINING	26.785 ± 43.815	19.903 ± 33.472	22.262 ± 72.089	11.440 ± 20.053	9.395 ± 16.694	23.937 ± 51.955

## Data Availability

The data used to support the findings of this study are included in the article.

## References

[1] Park H. N., Lim Y. J., Yi W. J., Han J. S., Lee S. P. (2018). A comparison of the accuracy of intraoral scanners using an intraoral *e*nvironment simulator. *Journal of Advanced Prosthodontics*.

[2] Cai H. X., Jia Q., Shi H. Y. (2020). Accuracy and precision evaluation of international standard spherical model by digital dental scanners. *Scanning*.

[3] Rotar R. N., Jivanescu A., Ille C. (2019). Trueness and precision of two intraoral scanners: a comparative in vitro study. *Scanning*.

[4] Mangano F., Mangano C., Margiani B., Admakin O. (2019). Combining intraoral and face scans for the design and fabrication of computer- assisted design/computer-assisted manufacturing (CAD/CAM) polyether-ether- ketone (PEEK) implant-supported bars for maxillary overdentures. *Scanning*.

[5] Lee J. J., Jeong I. I. D., Park J. Y., Jeon J. H., Kim J. H., Kim W. C. (2017). Accuracy of single-abutment digital cast obtained using intraoral and cast scanners. *Journal of Prosthetic Dentistry*.

[6] Patzelt S. B. M., Emmanouilidi A., Stampf S., Strub J. R., Att W. (2014). Accuracy of full-arch scans using intraoral scanners. *Clinical Oral Investigations*.

[7] Camardella L. T., Breuning H., de Vasconcellos Vilella O. (2017). Accuracy and reproducibility of measurements on plaster models and digital models created using an intraoral scanner. *Journal of Orofacial Orthopedics-Fortschritte Der Kieferorthopadie*.

[8] Jeong I. D., Lee J. J., Jeon J. H., Kim J. H., Kim H. Y., Kim W. C. (2016). Accuracy of complete-arch model using an intraoral video scanner: an in vitro study. *Journal of Prosthetic Dentistry*.

[9] Muallah J., Wesemann C., Nowak R. (2017). Accuracy of full-arch scans using intraoral and extraoral scanners: an in vitro study using a new method of evaluation. *International Journal of Computerized Dentistry*.

[10] Richert R., Goujat A., Venet L. (2017). Intraoral scanner technologies: a review to make a successful impression. *Journal of Healthcare Engineering*.

[11] Burde A. V., Gasparik C., Moldovan M., Baciu S., Cosma C. (2018). In vitro evaluation of accuracy of single dies captured by two intraoral digital scanners. *Materiale Plastice*.

[12] Gan N., Xiong Y. Y., Jiao T. (2016). Accuracy of intraoral digital impressions for whole upper jaws, including full dentitions and palatal soft tissues. *PLoS One*.

[13] Lim J. H., Park J. M., Kim M., Heo S. J., Myung J. Y. (2018). Comparison of digital intraoral scanner reproducibility and image trueness considering repetitive experience. *Journal of Prosthetic Dentistry*.

[14] Ardelean L. C., Rusu L. C., Stanciu S. G., Bueno J. M. (2020). Novel scanning characterization approaches for the accurate understanding and successful treatment of oral and maxillofacial pathologies. *Scanning*.

[15] Nedelcu R., Olsson P., Nyström I., Rydén J., Thor A. (2018). Accuracy and precision of 3 intraoral scanners and accuracy of conventional impressions: a novel in vivo analysis method. *Journal of Dentistry*.

[16] Nedelcu R., Olsson P., Nyström I., Thor A. (2018). Finish line distinctness and accuracy in 7 intraoral scanners versus conventional impression: an in vitro descriptive comparison. *BMC Oral Health*.

[17] Ahmed K. E., Whitters J., Ju X., Pierce S., MacLeod C., Murray C. (2016). A proposed methodology to assess the accuracy of 3D scanners and casts and monitor tooth wear progression in patients. *International Journal of Prosthodontics*.

[18] Wong K. Y., Esguerra R. J., Chia V. A. P., Tan Y. H., Tan K. B. C. (2018). Three-dimensional accuracy of digital static interocclusal registration by three intraoral scanner systems. *Journal of Prosthodontics*.

[19] Flügge T. V., Schlager S., Nelson K., Nahles S., Metzger M. C. (2013). Precision of intraoral digital dental impressions with iTero and extraoral digitization with the iTero and a model scanner. *American Journal of Orthodontics and Dentofacial Orthopedics*.

[20] Tomita Y., Uechi J., Konno M., Sasamoto S., Iijima M., Mizoguchi I. (2018). Accuracy of digital models generated by conventional impression/plaster-model methods and intraoral scanning. *Dental Materials Journal*.

[21] Imburgia M., Logozzo S., Hauschild U., Veronesi G., Mangano C., Mangano F. G. (2017). Accuracy of four intraoral scanners in oral implantology: a comparative in vitro study. *BMC Oral Health*.

[22] Duret F., Blouin J. L. (1986). Optical impressions in the computer-assisted design and fabrication of dental crowns. *Le Journal Dentaire du Québec*.

[23] van Noort R. (2012). The future of dental devices is digital. *Dental Materials*.

[24] Shimizu S., Shinya A., Kuroda S., Gomi H. (2017). The accuracy of the CAD system using intraoral and extraoral scanners for designing of fixed dental prostheses. *Dental Materials Journal*.

[25] Arakida T., Kanazawa M., Iwaki M., Suzuki T., Minakuchi S. (2018). Evaluating the influence of ambient light on scanning trueness, precision, and time of intra oral scanner. *Journal of Prosthodontic Research*.

[26] Logozzo S., Zanetti E. M., Franceschini G., Kilpelä A., Mäkynen A. (2014). Recent advances in dental optics - part I: 3D intraoral scanners for restorative dentistry. *Optics and Lasers in Engineering*.

[27] Jeon J. H., Choi B. Y., Kim C. M., Kim J. H., Kim H. Y., Kim W. C. (2015). Three-dimensional evaluation of the repeatability of scanned conventional impressions of prepared teeth generated with white- and blue-light scanners. *Journal of Prosthetic Dentistry*.

[28] Bocklet C., Renne W., Mennito A. (2019). Effect of scan substrates on accuracy of 7 intraoral digital impression systems using human maxilla model. *Orthodontics & Craniofacial Research*.

[29] Suh Y. S. (2019). Laser sensors for displacement, distance and position. *Sensors*.

[30] Kim J., Park J. M., Kim M., Heo S. J., Shin I. H., Kim M. (2016). Comparison of experience curves between two 3-dimensional intraoral scanners. *Journal of Prosthetic Dentistry*.

[31] Alyaman M., Abd-Raheem A., AlDeiri F. (2019). Design of an automated extraoral photogrammetry 3D scanner. *International Arab Journal of Information Technology*.

